# Quality of Evidence Supporting the Role of Acupuncture for the Treatment of Irritable Bowel Syndrome

**DOI:** 10.1155/2021/2752246

**Published:** 2021-12-15

**Authors:** Jinke Huang, Mengxiong Lu, Yijun Zheng, Jinxin Ma, Xiangxue Ma, Yitian Wang, Kunli Zhang, Fengyun Wang, Xudong Tang

**Affiliations:** ^1^Department of Gastroenterology, Xiyuan Hospital of China Academy of Chinese Medical Sciences, Beijing, China; ^2^Department of Gastroenterology, Peking University Traditional Chinese Medicine Clinical Medical School (Xiyuan), Beijing, China; ^3^China Academy of Chinese Medical Sciences, Beijing, China

## Abstract

**Objectives:**

To systematically collate, appraise, and synthesize the current evidence on acupuncture for irritable bowel syndrome (IBS).

**Methods:**

Systematic reviews (SRs)/meta-analyses (MAs) of acupuncture for IBS were searched in eight databases. For quality evaluation of the enrolled studies, Assessment of Multiple Systematic Reviews 2 (AMSTAR-2) was used for methodological quality, Preferred Reporting Item for Systematic Reviews and Meta-Analyses (PRISMA) for reporting quality, and Grading of Recommendations Assessment, Development, and Evaluation (GRADE) for evidence quality.

**Results:**

Ten studies were included in our review. According to AMSTAR-2, only one study met all the criteria and was rated as high methodological quality, and the rest were rated as low or very low methodological quality. According to the PRISMA checklist, most of the items were fully reported, with the exception of Q5 (protocol and registration), Q8 (search), and Q27 (funding). With the GRADE system, no outcome measure was rated as high quality.

**Conclusions:**

Acupuncture may be a promising therapy for IBS. However, this conclusion must be treated with caution since the quality of SRs/MAs providing evidence is generally low.

## 1. Introduction

Irritable bowel syndrome (IBS) is a functional gastrointestinal disorder characterized by recurrent abdominal pain accompanied by abnormal or altered defecation habits [1]. From country to country, the prevalence of IBS ranges from 1.1% to 45.0% [2], with global estimates of 11.2% [3] in Rome, 5.9% in China [4], and 7.1% in the United States [3]. This disorder not only has a marked negative impact on quality of life (QOL) and work productivity but also increases medical healthcare costs and imposes a huge socioeconomic burden [5, 6]. It is reported that the annual direct cost per patient due to IBS is estimated to be $348 to $8,750 and the indirect cost is $355 to $3,344 [7].

The pathophysiology of IBS is poorly understood and is currently thought to represent a complex interplay among the gut microbiota, mucosal immune system, impaired mucosal barrier function, visceral hypersensitivity, gut motility, and alterations in the gut-brain axis [8–10]. The conventional medication (CM) recommended to alleviate the symptoms include antispasmodics, fiber supplementation antidepressants, and probiotics [2, 8]. However, the effects are limited and accompanied by various side effects [11]. As a nonpharmacological treatment technique, acupuncture is believed to be beneficial to IBS based on the theory of the visceral hyperalgesia theory of the central nervous system.

Acupuncture is becoming more widely used, and the number of published systematic reviews (SRs) and meta-analyses (MAs) has increased, but the evidence they provide for acupuncture for IBS is not always consistent. SR/MA is considered the gold standard for assessing the effectiveness of clinical interventions; however, high-quality SRs/MAs can provide reliable evidence, while low-quality SRs/MAs might instead mislead clinical decision-making [12]. Thus, there may be a gap between evidence-based clinical implementation of acupuncture and its actual implementation in real-world dynamics. Clinical decision-making requires a comprehensive overview of the available evidence in order to identify potential benefits and harms of the intervention [13]. Within this framework, the overview of SRs/MAs is a relatively new approach, which aims to summarize and evaluate the strength of the evidence provided in multiple SRs/MAs [14]. By mapping the evidence in the real-world implementation field of acupuncture, an umbrella review will help draw a clear link between the need to address uncertainty and advancing clinical knowledge a priori [15]. Therefore, we conducted this study.

## 2. Methods

The Cochrane criteria and the statements of Preferred Reporting Item for Systematic Reviews and Meta-Analyses (PRISMA) [16] were followed to carry out this overview. The protocol was registered in PROSPERO (CRD42021228185).

### 2.1. Strategy for Search

PubMed, Cochrane Library, Web of Science, Embase, Chinese Scientific Journal Database, CNKI, VIP, and Wanfang were systematically searched from inception to July, 2021. Irritable bowel syndrome, acupuncture, systematic review, and meta-analyses were used as search key terms. A search strategy used for PubMed is shown in Table 1.

### 2.2. Criteria Used to Consider Studies

The studies that met the following criteria were included: (1) SRs/MAs based on randomized controlled trials; (2) the Rome I–IV criteria were adopted as diagnostic criteria for IBS; (3) the experimental intervention was acupuncture or a combination of acupuncture plus medications and the control intervention was Sham acupuncture or CM; and (4) outcome measures should be effective rate, recurrence rate, IBS symptom scores, IBS-QOL, and Symptom Severity Scale of IBS (IBS-SSS). The studies that met the following criteria were excluded: (1) duplicate publications; (2) updated SRs/MAs; (3) dissertations without peer review; and (4) the control intervention that included acupuncture.

### 2.3. Literature Selection and Data Extraction

Literature selection and data extraction were carried out by two independent authors, respectively. For literature selection, titles and abstracts were first screened and then, the full text of potentially relevant studies was further reviewed to determine eligibility. In addition to the outcomes of meta-analyses, data regarding the characteristics of the studies and subjects, details of the treatments, and methods of the SRs/MAs were extracted. Any discrepancies were resolved by discussion.

### 2.4. Quality Assessment

Quality assessment was carried out by two independent authors. The Assessment of Multiple Systematic Reviews 2 (AMSTAR-2) [17], PRISMA tool, and Grading of Recommendations Assessment, Development, and Evaluation (GRADE) [18] were used to evaluate the methodological quality, reporting quality, and evidence quality, respectively. Any discrepancies were resolved by discussion.

## 3. Results

### 3.1. Included Studies

**Figure 1:**
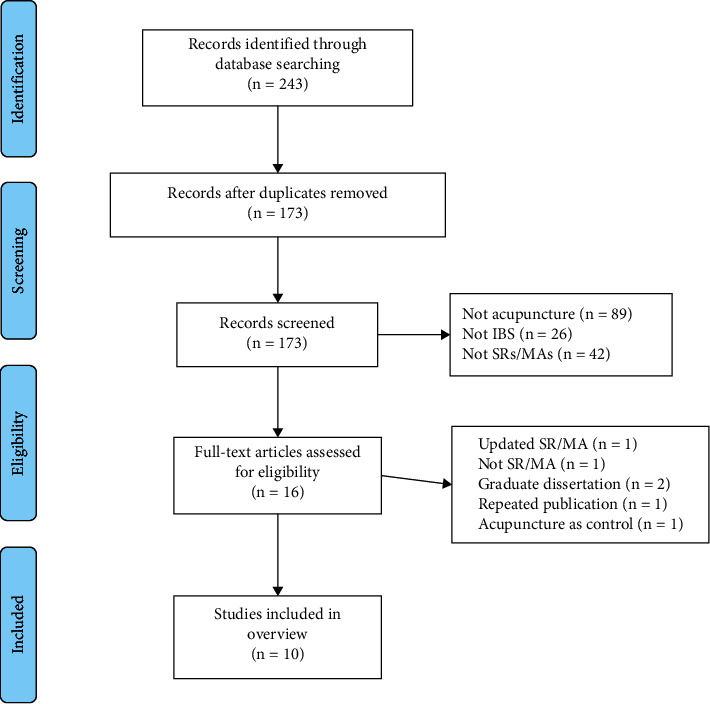
Literature screening flow chart.

As shown in Figure 1, the literature search identified 243 citations, and after removing the duplicates, 173 citations were further eliminated, 167 of which were excluded. Finally, 10 studies [19–28] met the inclusion criteria.

### 3.2. Study Characteristics

As shown in Table 2, 10 MAs published from 2010 to 2020 were enrolled in this overview. Half of these studies were published in English, with the number of trails ranging from 6 to 41 and the subjects ranging from 664 to 3440. The experimental intervention was mainly acupuncture or a combination of acupuncture plus medications, and the control intervention was mainly Sham acupuncture or CM characteristics.

### 3.3. Quality Assessment

#### 3.3.1. Methodological Appraisal

**Figure 2:**
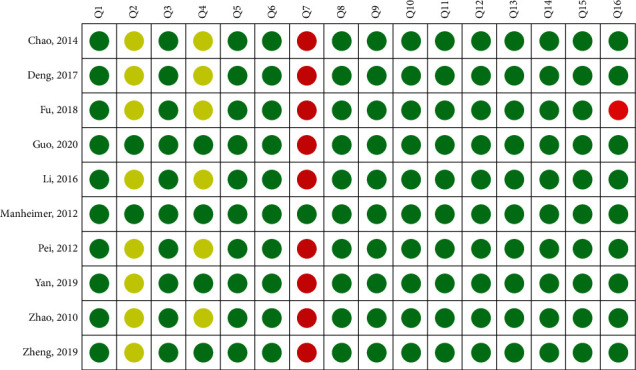
Summary of the AMSTAR-2 assessments.

**Figure 3:**
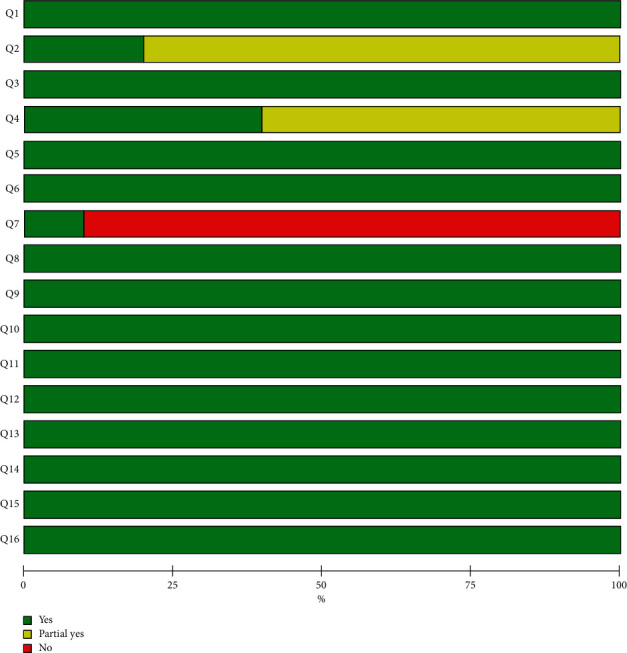
Graphical representation of the AMSTAR-2 assessments.

According to AMSTAR-2, only one review met all items and was rated as high methodological quality, and the rest were rated as low or critically low methodological quality. Key items affecting the methodological quality were item 2 (established protocol), item 4 (comprehensive search strategy), and item 7 (a list of excluded trails). Further details are shown in Figures 2 and 3.

#### 3.3.2. Reporting Quality Appraisal

According to PRISMA checklists, most of the items were fully reported in these included reviews, with the exception of Q5 (protocol and registration), Q8 (search), and Q27 (funding). Further details are given in Table 3.

#### 3.3.3. Evidence Quality Classification

25 outcome indicators regarding the effects of acupuncture for IBS were extracted from the included studies. With GRADE, 12 outcome indicators were rated as moderate quality and the rest were rated as low or critically low quality. The risk of bias, imprecision, inconsistency, and publication bias were the main reasons for evidence degradation (Table 4).

### 3.4. Description of Efficacy

#### 3.4.1. Effect of the Interventions

Relative effects of the outcome indicators regarding the effectiveness of acupuncture for IBS are shown in Table 4. Two studies [20, 23] compared the effects of acupuncture and Sham acupuncture, and reportedly no statistically significant difference was found in effective rate, IBS-QOL, or IBS-SSS. Nine studies [19, 20, 22–28] compared the effects of acupuncture and CM, and results revealed that patients receiving acupuncture therapy showed a greater improvement in effective rate, recurrence rate, weekly defecation, IBS symptom scores, IBS-QOL, and IBS-SSS than patients receiving CM. One study [21] compared the effects of acupuncture plus Chinese herbal medicine and CM, and results revealed that patients receiving combination therapy reported a significantly greater improvement in effective rate and abdominal pain than patients receiving CM.

#### 3.4.2. Safety of the Interventions

One study [19] reported the meta-analysis results in adverse effects, and no statistically significant difference was found between patients treated with acupuncture and CM.

## 4. Discussion

Treatment of IBS focuses on symptom management to maintain daily functioning and improve QOL. However, due to significant side effects of prescribed medications, some sufferers do not take multiple CM but instead turn to complementary and alternative therapies for remedy [11, 29]. A number of SRs/MAs have investigated the efficacy of acupuncture for IBS patients. The purpose of this study was to systematically collate, appraise, and synthesize the evidence published in recent years.

Ten SRs/MAs regarding to the efficacy of acupuncture for IBS were finally included. From the meta-analysis results of these studies, patients reported that acupuncture had a greater benefit on IBS symptoms than CM. However, these findings must be considered cautiously, given the limitations on methodological quality, reporting quality, and evidence quality of the included studies. According to AMSTAR-2 and PRISMA checklists, most of (80%) the included studies did not establish a protocol, which could undermine the rigor of the study and increase the risk of bias. For literature search, 60% studies only provided the search keywords but no specific search strategies, which could lead to publication bias and undermined the credibility of the results. Moreover, 90% studies did not provide the lists of excluded trails, which may undermine the transparency of the study process. According to the GRADE tool, no outcome indicators provided high-quality evidence, indicating that the meta-analyses results of the included studies may differ from the true results. The risk of bias for the enrolled trails of the included studies was the main reason for evidence degradation. Further analyses found common limitations of the enrolled trails as follows: only randomization was mentioned without the randomization method; the allocation was not concealed; and only single blinding was implemented. Therefore, the basic factor leading to the decline in the quality of evidence was the low methodological quality of the enrolled trails. It was believed that well-designed and implemented randomized controlled trials were considered to be the gold standard to avoid the risk of bias [30]. Furthermore, almost all of the included SRs/MAs indicated that acupuncture seemed to have a significant clinical efficacy for IBS; however, most authors did not wish to draw clear conclusions due to low methodological quality or the small size of the enrolled trails.

The action mechanism of acupuncture for IBS includes regulating the gastrointestinal motility, reducing visceral hypersensitivity, regulating the brain-intestine axis, reducing low-level intestinal mucosal inflammation, promoting intestinal microflora balance, and adjusting psycho-psychological status [31]. IBS is a gastrointestinal disorder in which intestinal spasm causes abdominal pain, hypermotility leads to diarrhea, and hypomotility leads to constipation. Thus, for the purpose of treatment, IBS can be divided into three types: constipation-predominant, diarrhea-predominant, or mixed [32]. Animal experiments revealed that acupuncture stimulation of IBS-D model rats effectively improved diarrhea symptoms in rats, and it was found that the mRNA and protein expression of APQ8 in the rat colon tissue was reduced, while the protein expression of VIP was increased [33]. For patients with IBS-C, electroacupuncture stimulation of Zusanli can promote contraction of the patient's colon ends and accelerate colonic transit, which in turn improves constipation symptoms [34]. These results suggest that acupuncture has a bidirectional regulatory effect on intestinal motility in IBS patients. Furthermore, EA intervention can ameliorate the fecal property in IBS-C rats, which may be associated with its function in inhibiting the expression of colonic CGRP and SP proteins [35]. Visceral hypersensitivity is considered an important pathological mechanism in the development of IBS. It is reported that EA can alleviate visceral hypersensitivity in IBS-D and IBS-C rats by regulating the expression level of TRPV1 in the colon [35, 36]. The brain-gut axis was a complex, bidirectional signaling system between the central nervous system and the gastrointestinal system. It is reported that acupuncture could improve intestinal motility and visceral sensitivity by modulating brain-gut peptide levels in the central nervous system, gut, and blood [31]. Furthermore, electroacupuncture decreases 5-HT and CGRP, increases NPY in the brain-gut axis in rat models of IBS-D [37], and increases the number of neurons in the myenteric plexus of IBS-C rats [38]. Posttraumatic stress disorder (PTSD) is thought to be associated with IBS and is a common comorbidity [39]. It is reported that acupuncture can affect the autonomic nervous system, and the prefrontal as well as limbic brain structures, enabling it to relieve the symptoms of PTSD [40]. Activation of the immune system was strongly associated with IBS, and acupuncture could downregulate the expression of serum IL-18, TNF-*α*, and IL-23 in IBS patients, thus playing an immunoregulatory role [41]. The overgrowth of intestinal flora may be an important factor in the induction of IBS [42]. It is reported that acupuncture treatment may modulate intestinal bacteria and the psychological state tends to balance to relieve the symptoms of IBS [31, 43]. However, there is still a lack of evidence on the regulation of intestinal microbiota in IBS through the use of acupuncture.

This overview would provide some useful information on unique treatments in clinical practice for physicians in the management of IBS, thus providing more treatment options for IBS patients. However, we found that the majority of the included reviews were of poor quality, which could result in them having low credibility. Furthermore, the AMSTAR-2 tool, PRISMA checklist, and the GRADE system are highly subjective. Thus, different reviewers may have their own independent judgments on the evaluation results. Even with two independent reviewers in this study, subjective factors or errors cannot be completely eliminated. Finally, there is limited evidence for the efficacy of acupuncture for IBS subtypes, especially IBS-C. Further clinical and mechanistic studies of acupuncture for IBS subtypes are still necessary.

## 5. Conclusion

Acupuncture may be a promising treatment for IBS, and it could be used as an adjunct in clinical settings to improve efficacy. However, this conclusion must be treated with caution since the quality of SRs/MAs providing evidence is generally low.

## Figures and Tables

**Table 1:** Search strategy for the PubMed database.

Query	Search term
#1	Irritable bowel syndrome [Mesh]
#2	Irritable bowel syndrome [Title/abstract] OR irritable colon syndrome [Title/abstract] OR irritable colon [Title/abstract] OR gastrointestinal syndrome [Title/abstract] OR colon spasm [Title/abstract] OR allergic colitis [Title/abstract] OR colon allergy [Title/abstract] OR IBS [Title/abstract]
#3	#1 OR #2
#4	Acupuncture [Mesh]
#5	Acupuncture [title/abstract] OR pharmacoacupuncture [title/abstract] OR acupotomy [title/abstract] OR acupotomies [title/abstract] OR pharmacopuncture [title/abstract] OR needle [title/abstract] OR needling [title/abstract] OR dry-needling [title/abstract] OR body-acupuncture [title/abstract] OR electroacupuncture [title/abstract] OR electro-acupuncture [title/abstract] OR auricular acupuncture [title/abstract] OR warm needle [title/abstract]
#6	#4 OR #5
#7	Meta-analysis as topic [mesh]
#8	Systematic review [title/abstract] OR meta-analysis [title/abstract] OR meta-analyses [title/abstract]
#9	#7 OR #8
#10	#3 AND #6 AND #9

**Table 2:** Baseline characteristics of included reviews.

Studies	Country	Trials (subjects)	Experimental Intervention	Control Intervention	Quality assessment	Meta-analyses	Results summary
Guo et al. [19]	China	31 (3234)	AT	CM	Cochrane criteria	Yes	Acupuncture was an effective and safe therapy for IBS.
Zheng et al. [20]	China	41 (3440)	AT, AT + CM	Sham AT, CM	Cochrane criteria	Yes	The effect of acupuncture on IBS was better than that of CM, which could be used as an adjuvant therapy in clinical practice.
Yan et al. [21]	China	21 (1834)	AT + CHM	CM; CHM	Cochrane criteria	Yes	The combination of acupuncture and Chinese herbal medicine was effective and safe in the treatment of IBS.
Chao and Zhang [22]	China	6 (664)	AT	Sham AT, CM	Jadad	Yes	Acupuncture was significant in relieving the symptoms of IBS.
Manheimer et al. [23]	United States	17 (1806)	AT	Sham AT, CM	Cochrane criteria	Yes	The effect of acupuncture on IBS was better than that of CM, which could be used as an adjuvant therapy in clinical practice.
Fu and Jiang [24]	China	23 (1685)	AT	CM; AT + CM	Jadad	Yes	Acupuncture therapy was superior to conventional CM in the treatment of IBS.
Deng et al. [25]	China	17 (1333)	AT; AT + CM	CM; Sham AT + CM	Jadad	Yes	Acupuncture for IBS was superior to conventional treatment, which could improve the clinical symptoms and reduce the recurrence rate of patients.
Li et al. [26]	China	12 (715)	AT	CM	Cochrane criteria	Yes	The evidence of this study was not sufficient to prove that the efficacy of acupuncture was better than CM.
Pei et al. [27]	China	11 (969)	AT; AT + CM	CM; Sham AT + CM	Cochrane criteria	Yes	Acupuncture for IBS was better than the CM treatment.
Zhao et al. [28]	China	10 (810)	AT	CM	Jadad	Yes	The effect of acupuncture on IBS was superior to that of western medicine.

AT: acupuncture therapy; CHM: Chinese herbal medicine.

**Table 3:** Results of the PRISMA checklists.

Section/topic	Items	Guo, 2020	Zheng, 2019	Yan, 2019	Chao, 2014	Manheimer, 2012	Fu, 2018	Deng, 2017	Li, 2016	Pei, 2012	Zhao, 2010	Compliance (%)
Title	Q1. Title	Y	Y	Y	Y	Y	Y	Y	Y	Y	Y	100
Abstract	Q2. Structured summary	Y	Y	Y	Y	Y	Y	Y	Y	Y	Y	100
Introduction	Q3. Rationale	Y	Y	Y	Y	Y	Y	Y	Y	Y	Y	100
	Q4. Objectives	Y	Y	Y	Y	Y	Y	Y	Y	Y	Y	100
Methods	Q5. Protocol and registration	Y	N	N	N	Y	N	N	N	N	N	20
	Q6. Eligibility criteria	Y	Y	Y	Y	Y	Y	Y	Y	Y	Y	100
	Q7. Information sources	Y	Y	Y	Y	Y	Y	Y	Y	Y	Y	100
	Q8. Search	Y	Y	Y	PY	Y	PY	PY	PY	PY	PY	40
	Q9. Study selection	Y	Y	Y	Y	Y	Y	Y	Y	Y	Y	100
	Q10. Data collection process	Y	Y	Y	Y	Y	Y	Y	Y	Y	Y	100
	Q11. Data items	Y	Y	Y	Y	Y	Y	Y	Y	Y	Y	100
	Q12. Risk of bias in individual studies	Y	Y	Y	Y	Y	Y	Y	Y	Y	Y	100
	Q13. Summary measures	Y	Y	Y	Y	Y	Y	Y	Y	Y	Y	100
	Q14. Synthesis of results	Y	Y	Y	Y	Y	Y	Y	Y	Y	Y	100
	Q15. Risk of bias across studies	Y	Y	Y	Y	Y	Y	Y	Y	Y	Y	100
	Q16. Additional analyses	Y	Y	Y	Y	Y	Y	Y	Y	Y	Y	100
Results	Q17. Study selection	Y	Y	Y	Y	Y	Y	Y	Y	Y	Y	100
	Q18. Study characteristics	Y	Y	Y	Y	Y	Y	Y	Y	Y	Y	100
	Q19. Risk of bias within studies	Y	Y	Y	Y	Y	Y	Y	Y	Y	Y	100
	Q20. Results of individual studies	Y	Y	Y	Y	Y	Y	Y	Y	Y	Y	100
	Q21. Synthesis of results	Y	Y	Y	Y	Y	Y	Y	Y	Y	Y	100
	Q22 Risk of bias across studies	Y	Y	Y	Y	Y	Y	Y	Y	Y	Y	100
	Q23. Additional analysis	Y	Y	Y	Y	Y	Y	Y	Y	Y	Y	100
Discussion	Q24. Summary of evidence	Y	Y	Y	Y	Y	Y	Y	Y	Y	Y	100
	Q25. Limitations	Y	Y	Y	Y	Y	Y	Y	Y	Y	Y	100
	Q26. Conclusions	Y	Y	Y	Y	Y	Y	Y	Y	Y	Y	100
Funding	Q27. Funding	Y	Y	Y	Y	Y	N	Y	Y	Y	Y	90

**Table 4:** Certainty of evidence quality.

Studies	Treatments	Outcomes	Limitations	Inconsistency	Indirectness	Imprecision	Publication bias	Relative effect (95% CI)	Quality
Guo et al. [19]	AT versus CM	Weekly defecation	−1	0	0	0	0	SMD, −0.29 (−0.49, −0.08)	M
		IBS symptom scores	−1	0	0	0	0	SMD, −1.17 (−1.42, −0.93)	M
		IBS-QOL	−1	0	0	−1	0	SMD 2.37 (1.94, 2.80)	L
		IBS-SSS	−1	0	0	0	0	SMD −0.75 (−1.04, −0.47)	M
		Effective rate	−1	0	0	0	0	RR 1.25 (1.18, 1.32)	M
		Recurrence rate	−1	0	0	−1	0	RR 0.43 (0.28, 0.66)	L
		Adverse effects	−1	0	0	−1	0	RR 0.59 (0.12, 2.90)	L
Zheng et al. [20]	AT versus Sham AT	Effective rate	−1	0	0	0	0	RR 1.22 (1.01, 1.47)	M
		IBS-QOL	−1	0	0	0	0	SMD −0.10 (−0.31, 0.11)	M
	AT versus CM	Effective rate	−1	0	0	0	0	RR 1.17 (1.12, 1.23)	M
		IBS symptom scores	−1	−1	0	0	0	SMD −1.16 (−1.61, −0.71)	L
		IBS-QOL	−1	0	0	−1	0	SMD 0.75 (0.34, 1.16)	L
Yan et al. [21]	AT + CHM versus CM	Effective rate	−1	0	0	0	0	RR 1.29 (1.24, 1.35)	M
		Abdominal pain	−1	−1	0	0	0	SMD −0.45 (−0.72, −0.17)	L
Chao and Zhang [22]	AT versus CM	Effective rate	−1	0	0	0	0	RR 1.75 (1.24, 2.46)	M
Manheimer et al. [23]	AT versus Sham AT	IBS-SSS	−1	0	0	−1	0	SMD −0.11 (−0.35, 0.13)	L
		IBS-QOL	−1	0	0	−1	0	SMD −0.03 (−0.27, 0.22)	L
	AT versus CM	Effective rate	−1	0	0	0	0	RR 1.28 (1.12, 1.45)	M
Fu and Jiang [24]	AT versus CM	Effective rate	−1	0	0	0	0	RR 1.20 (1.15, 1.25)	M
Deng et al. [25]	AT versus CM	Effective rate	−1	0	0	0	0	OR 3.92 (2.83, 5.43)	M
		Recurrence rate	−1	0	0	−1	0	OR 0.22 (0.12, 0.41)	L
Li et al. [26]	AT versus CM	Recurrence rate	−1	0	0	−1	−1	RR 0.49 (0.35, 0.68)	CL
		Effective rate	−1	0	0	0	−1	RR 1.17 (1.08, 1.26)	L
Pei et al. [27]	AT versus CM	Effective rate	−1	−1	0	0	0	RR 1.27 (1.09, 1.49)	L
Zhao [28]	AT versus CM	Effective rate	−1	0	0	0	−1	RR 1.28 (1.20, 1.38)	L

## Data Availability

All analyses were based on previously published studies.

## Abbreviations

IBS: Irritable bowel syndrome
SR: Systematic review
MA: Meta-analysis
AMSTAR-2: Assessment of Multiple Systematic Reviews 2
PRISMA: Preferred Reporting Item for Systematic Reviews and Meta-Analyses
GRADE: Grading of Recommendations, Assessment, Development, and Evaluation
QOL: Quality of life
SSS: Symptom Severity Scale
CM: Conventional medication.
